# The Graz Malnutrition Screening (GMS): a new hospital screening tool for malnutrition

**DOI:** 10.1017/S0007114515004924

**Published:** 2015-12-14

**Authors:** Regina E. Roller, Doris Eglseer, Anna Eisenberger, Gerhard H. Wirnsberger

**Affiliations:** 1 Department of Internal Medicine, Medical University of Graz, Auenbruggerplatz 15, A-8036 Graz, Austria; 2 Center of Dietetics and Applied Nutrition, General Hospital Graz, Auenbruggerplatz 21, 8036 Graz, Austria

**Keywords:** Malnutrition, Risk screening, Adults, Hospital nutrition

## Abstract

Despite the significant impact of malnutrition in hospitalised patients, it is often not identified by clinical staff in daily practice. To improve nutritional support in hospitals, standardised routine nutritional screening is essential. The Graz Malnutrition Screening (GMS) tool was developed for the purpose of malnutrition risk screening in a large hospital setting involving different departments. It was the aim of the present study to validate the GMS against Nutritional Risk Screening (NRS) and Mini Nutritional Assessment-short form (MNA-sf) in a randomised blinded manner. A total of 404 randomly selected patients admitted to the internal, surgical and orthopaedic wards of the University Hospital Graz were screened in a blinded manner by different raters. Concurrent validity was determined by comparing the GMS with the NRS and in older patients (70+ years) with the MNA-sf additionally. According to GMS, 31·9 or 28·5 % of the admitted patients were categorised as at ‘risk of malnutrition’ (depending on the rater). According to the reference standard of NRS, 24·5 % of the patients suffered from malnutrition. Pearson’s *r* values of 0·78 compared with the NRS and 0·84 compared with the MNA showed strong positive correlations. Results of accuracy (0·85), sensitivity (0·94), specificity (0·77), positive predictive value (0·76) and negative predictive value (0·95) of GMS were also very high. Cohen’s *κ* for internal consistency of the GMS was 0·82. GMS proves to be a valid and reliable instrument for the detection of malnutrition in adult patients in acute-care hospitals.

Malnutrition is highly prevalent in hospitalised patients, varying from 20 to 60 % upon admission. Prevalence estimation strongly depends on the underlying definition of malnutrition and pre-defined evaluation parameters^(^ ^1^ ^–^ ^6^ ^)^. The European Society for Clinical Nutrition and Metabolism (ESPEN) defines malnutrition as a state of nutrition in which a deficiency or excess (or imbalance) of energy, protein and other nutrients causes measurable adverse effects on tissue/body form (body shape, size and composition) and function, as well as clinical outcome^(^ ^7^ ^)^. The American Society on Parenteral and Enteral Nutrition (ASPEN) broadens the approach. According to the ASPEN guideline, malnutrition is defined as ‘an acute, sub-acute or chronic state of nutrition, in which varying degrees of over-nutrition or under-nutrition with or without inflammatory activity have led to a change in body composition and diminished function’^(^ ^8^ ^)^.

The goal of nutritional screening is to identify any specific nutrition risk(s). Indeed, the above-mentioned definitions are consistent with the Joint Commission on Accreditation of Healthcare Organisation’s interpretation of a ‘screen’ as an instrument used to determine whether additional information (from an assessment) is required to warrant an intervention^(^ ^9^ ^)^. An International Consensus Guideline Committee has proposed an approach to diagnosing malnutrition in adults based on aetiology, thus integrating the present understanding of inflammatory responses to disease and trauma^(^ ^10^ ^,^ ^11^ ^)^. At present, there are numerous screening tools for malnutrition in adults cited in the literature. The ESPEN as well as the ASPEN recommendations also include guidelines on how to evaluate malnutrition risk in the hospital setting^(^ ^7^ ^,^ ^8^ ^)^. However, guidelines cannot account for every variation in circumstances. Hospitals therefore must always exercise professional judgement and look for applications feasible for their needs, based on internationally agreed core components within the definition of malnutrition. One of the major challenges in hospital-care settings is insufficient knowledge and low commitment among nurses and physicians regarding the topic of malnutrition, which results in an insufficient focus on nutritional aspects of care^(^ ^12^ ^–^ ^14^ ^)^. Therefore, it is a great challenge to implement nutritional guidelines in hospitals^(^ ^15^ ^–^ ^19^ ^)^.

The Medical University of Graz (MUG) comprises forty clinical and non-clinical institutions. It is closely associated with the General Hospital Graz, which is among the largest hospitals in Europe and is a top-quality institution offering all fields of human medicine. The University Hospital Graz currently accommodates all medical disciplines within seventy organisational units and 1578 acute-care beds. Approximately 385 000 patients are being treated in the clinics annually. As for the specialty of internal medicine, approximately 70 % of the patients admitted to the wards are older than 65 years. In admitted patients older than 75 years, the sex ratio of female:male is approximately 2:1 with a further increasing tendency towards women with increasing age.

It became essential to establish a malnutrition risk screening tool that addresses internationally recommended quality standards, is easy to handle in daily routine practice and that is highly sensitive in detection of individual malnutrition risk independent of sex, age or diagnosis at admission. Since 1997, a multi-disciplinary nutrition team, comprised of dietitians, medical doctors, pharmacists, nurses and staff from the local hospital management, stepped forward to implement a risk screening tool into the locally established hospital information technology (IT) platform (MEDOCS™) to improve compliance of healthcare professionals towards malnutrition risk screening on a routine basis. This screening instrument was primarily based on the tool of subjective global assessment (SGA), which initially had been dedicated and validated for surgical patients^(^ ^20^ ^,^ ^21^ ^)^. However, to address special needs of a big university hospital, the screening tool had to be adapted according to patients’ characteristics in the local setting. In terms of novelty, this malnutrition risk screening tool is based on electronic documentation and may be connected to any hospital IT platform. The Graz Malnutrition Screening (GMS) also has other advantages compared with currently available malnutrition risk screening tools. It has high user-friendliness and involves dietitians in an automated manner if malnutrition risk is detected in a patient who had been screened by the staff on the wards. GMS therefore assures the multi-professional approach and quality control. Using the GMS, we implemented effective governance via the establishment of a multi-disciplinary nutrition steering committee at each facility with representation from medical, food services, nursing, dietetic, pharmacy and speech pathology staff.

It was the aim of the current study to validate the GMS in a blinded randomised controlled trial against the gold standard of malnutrition risk screening in hospitals recommended by ESPEN, the Nutritional Risk Screening (NRS) tool^(^ ^22^ ^)^. In addition, the GMS was compared with the Mini Nutritional Assessment-short form (MNA-sf), which is recommended for malnutrition risk screening for people older than 65 years^(^ ^23^ ^,^ ^24^ ^)^.

## Methods

**Fig. 1:**
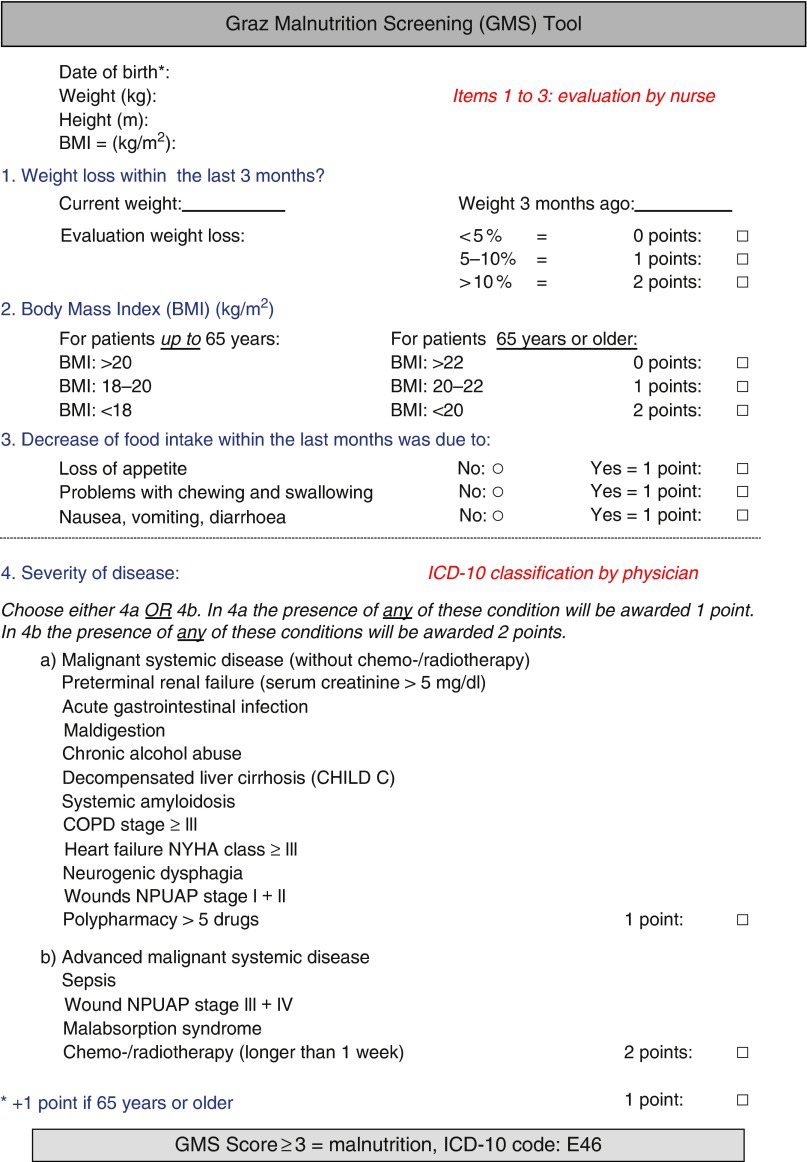
The Graz Malnutrition Screening (GMS) risk translated into English language. Items 1–3 are obtained from nursing staff. Item 4, which includes rating of disease, is completed by physicians. A total score of more than 3 points indicates ‘risk of malnutrition’. Information is gathered in the hospital software. In terms of positive screening, information is transferred to dietitians and assessment is performed and clinical nutritional intervention is started whenever indicated. ICD, International Classification of Diseases.

**Fig. 2:**
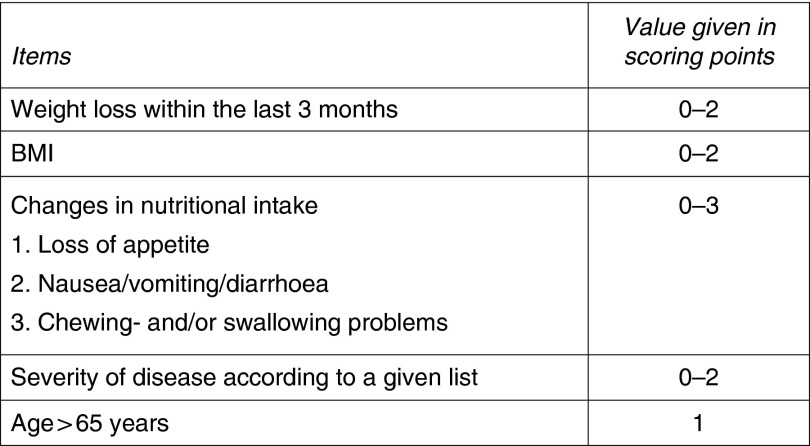
The Graz Malnutrition Screening consists of five different scoring categories.

The structure of the GMS is based on interdisciplinary work within the wards. The first part of screening, including BMI and dietary and stool patterns, is completed by nurses. Medical doctors treating patients in the wards (Fig. 1) classify risk of malnutrition according to underlying diseases^(^ ^25^ ^–^ ^33^ ^)^. It seems noteworthy that aged patients account for a large number of admissions at our university hospital. On the basis of recently developed knowledge regarding age as an independent risk factor for malnutrition and given local hospital challenges of acute medical care, it was decided that age >65 years is handled as an ‘independent risk factor’ in the screening tool accounting for 1 point on the scoring scale. The risk screening tool is designed so that a score of 3 or more signifies ‘risk of malnutrition’ (Fig. 2). All data collected during screening are stored within the electronic medical patient record. In the case of a positive screening, the patient record is forwarded to the dietitian. In accordance with legal regulation and local standard operating procedures, the dietitian is then integrated into the individual care process. If diagnosis of malnutrition is confirmed by consecutive assessments, the diagnosis ‘malnutrition’ (International Classification of Diseases (ICD) 10, code: E46) is automatically transferred to the patient’s electronic record.

### Study design

For validation of sensitivity and specificity of the GMS, a cross-sectional study design was chosen. Screening was performed using three screening pathways: routinely performed GMS by the ward team (listed as rater 1), GMS by a trained dietitian using the GMS (listed as rater 2) and NRS and MNA-sf performed by a study dietitian (listed in legends of figures as rater 3). All screenings were performed in a blinded manner. Within the study cohort, data were collected by pencil and paper in the wards and transformed into the study data file collectively by a study nurse.

### Recruitment of patients

Ethics approval was obtained from the independent Ethical Review Committee of the MUG. All patients admitted to the internal ward, the surgical ward or the orthopaedic ward between December 2013 and March 2014 were screened for study participation within the recruitment tool. Only patients able to provide informed consent were eligible for recruitment. Potential candidates for study participation were approached by the study team in a compliance talk and were also additionally provided written information. Patients confirmed their participation by signing an informed consent document. In the case where patients could not be met by the team – for example, due to procedures taken or length of stay – the electronic randomising programme provided substitute patients within the same age range.

### Data collection

For measuring height to the nearest 0·5 cm, a stadiometer (Seca 206 Bodymeter™; SECA GmbH & Co.) fixed to the wall was used. Weight was measured to the nearest 0·1 kg using ward- or clinic-based medical, calibrated scales. The percentage of weight loss in the last 3 months and nutritional intake were documented from the patient’s history routinely. Acute and chronic diseases were scored and documented by physicians at the ward. To determine inter-rater reliability of the GMS, patients were screened by two dietitians in a blinded and independent manner. Within 4-h, both raters visited the same patient and conducted the GMS. For measuring concurrent validity of the GMS, the NRS was used as a gold standard. The MNA-sf was conducted additionally in patients older than 70 years by a study dietitian working in a blinded manner from the rest of the study team.

### Statistics

Statistical analysis was performed using SPSS version 21.0 for Windows (SPSS). The number of patients needed to be screened to reach the level of significance had been estimated by the local statistician for the end points described. The cut-off value to prove statistical significance had been estimated at 400 participants. Baseline characteristics were analysed using descriptive statistics. Inter-rater reliability was tested with Cohen’s *κ* and percentage agreement (PA). *κ* Values were interpreted derived from Landis & Koch^(^ ^34^ ^)^. *κ* Values under 0·2 were defined as ‘slight agreement’, *κ* values between 0·21 and 0·4 were considered as ‘fair agreement’, *κ* values between 0·41 and 0·6 indicated ‘moderate’ and *κ* values between 0·61 and 0·80 a substantial agreement. *κ* Values>0·8 were considered as almost perfect agreement. Concurrent validity was determined matching the GMS and the NRS results of older patients (70+ years) with individual MNA-sf data using Pearson’s correlation. Furthermore, sensitivity, specificity, accuracy and positive/negative predictive values (PPV/NPV) were calculated. Internal consistency was estimated by calculation of Cronbach’s *α*.

## Results

**Fig. 3:**
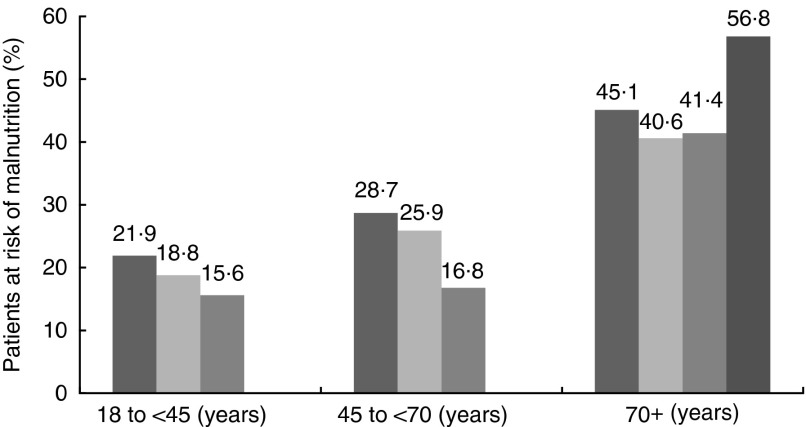
Prevalence of risk of malnutrition among all patients in different age groups tested with three screening tools used by different teams in a blinded manner. Differences between scores of different screening tools were not statistically significant. GMS 1, Graz Malnutrition Screening Rater 1 (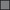); GMS 2, Graz Malnutrition Screening Rater 2 (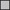); NRS, Nutritional Risk Screening Rater 3. 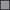, Nutritional Risk Screening Rater; 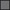, Mini Nutritional Assessment.

**Fig. 4:**
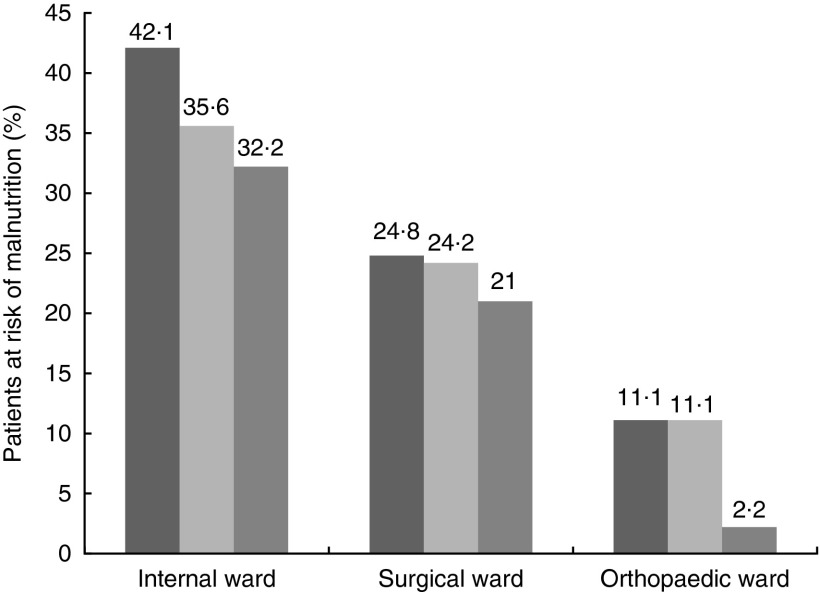
Percentage of patients identified to be at risk for malnutrition using the Graz Malnutrition Screening (GMS) and the Nutritional Risk Screening (NRS, 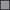). GMS 1 (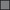), Graz Malnutrition Screening Rater 1; GMS 2 (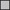), Graz Malnutrition Screening Rater 2; NRS, Nutritional Risk Screening Rater 3. All data are grouped for wards of assignment.

A total of 407 randomly selected patients, admitted to the internal medicine, surgical or orthopaedic wards were screened by the study team; three patients refused to participate. Finally, 404 patients were recruited and screened according to the protocol; 183 (45·3 %) participants were female and 221 (54·7 %) were male. Median patient age was 61 (18–93) years. A total of 128 patients were matched in age-group one (18–44 years), 143 in age-group two (45–69 years) and 133 in age-group three (70+ years). Risk of malnutrition in the overall cohort varied between age groups. There was an increasing prevalence of risk of malnutrition with age (Fig. 3). No statistically significant difference could be found for prevalence of malnutrition according to the screening tool used (Fig. 4).

Table 1 shows cross-tabulation for comparison of concurrent validity of the GMS and NRS applied in all patients enrolled. The Pearson’s correlation of the GMS scores and the NRS scores was *r* 0·78. The determination coefficient *r* ^2^ was 0·61. Out of overall 404 patients, there were fifty disagreements between tools, with forty out of fifty subjects’ risk being overestimated by GMS (Table 1). Using dichotomous variables (at risk for malnutrition/not malnourished) and presenting results as a cross-tabulation, accuracy of the GMS was 0·88 according to the NRS. Sensitivity (0·87) and specificity (0·90) were very high as well as PPV (0·96). The NPV score was (0·69). Correlation coefficient was even higher with 0·84 and *r* ^2^ was 0·71. Results for accuracy (0·85), sensitivity (0·94), specificity (0·77), PPV (0·76) and NPV (0·95) were also very high.

**Table 1:** Cross-tabulation of malnutrition risk in internal, surgical and orthopaedic patients according to the Graz Malnutrition Screening (GMS) and the Nutritional Risk Screening (NRS)

		NRS		
		Not at risk for malnutrition	At risk of malnutrition or malnourished	Total
GMS				
Not at risk		265	10	275
At risk		40	89	129
Total		305	99	404

Comparison of GMS and MNA-sf results indicated nineteen disagreements between the two tools with sixteen out of nineteen older patients’ risk underestimated by GMS compared with MNA (Table 2). It should be noted that the results of the MNA are divided into three categories (not malnourished, risk of malnutrition, malnourished), whereas the GMS consists of two categories (not malnourished, risk of malnutrition). Therefore, patients at risk of malnutrition and malnourished patients according to the MNA were summarised into one category.

**Table 2:** Cross-tabulation of malnutrition risk in older internal, surgical and orthopaedic patients according to the Graz Malnutrition Screening (GMS) and the Mini Nutritional Assessment-short form (MNA-sf)

		MNA-sf		
		Not at risk for malnutrition	At risk of malnutrition or malnourished	Total
GMS				
Not at risk		51	16	67
At risk		3	55	58
Total		54	71	125

### Inter-rater reliability and internal consistency

*κ* Values, PA and standard error of measurement for the single items as well as for the total scale are shown in Table 3. *κ* Values of the agreement between raters and reliability of single items within the GMS screening tool varied between 0·6 and 1·00. The *κ* value of the total scale was 0·82. PA was between 86·4 and 100 %, whereas PA of the total scale was 94·8 %; 100 % agreement was reached in the items BMI, severity of disease and age. Overall internal consistency Cronbach’s *α* was 0·57 with a range for the corrected item total.

**Table 3:** Agreement between two raters using the Graz Malnutrition Screening in 404 patients (Mean values with their standard errors)

	Cohen’s *κ*	PA	sem
Weight loss within the last 3 months	0·812	94·1	0·04
BMI (kg/m^2^)	1·000	100	0·00
Changes in nutritional intake			
Loss of appetite	0·631	86·4	0·05
Problems with chewing or swallowing	0·604	93·1	0·07
Nausea/vomiting/diarrhoea	0·614	89·9	0·05
Severity of disease	1·000	100	0·00
Age (years)	1·000	100	0·00
Total scale	0·824	94·8	0·03

PA, percentage agreement

### Malnutrition according to allocation of patients

According to the GMS, 129 patients (31·9 %) and 115 patients (28·5 %), respectively, were classified as ‘at risk for malnutrition’. Numbers at risk for malnutrition detected by the NRS were slightly but not statistically significantly lower (Fig. 3). Percentages of patients at risk for malnutrition older than 70 years were slightly different, depending on the rater (Fig. 3). MNA-sf detected risk of malnutrition with a slightly higher sensitivity. All differences were not statistically significant (data not shown).

## Discussion

According to Ljungqvist & de Man^(^ ^35^ ^)^, thirty-three million people are at risk of under-nutrition in Europe. Furthermore, a quarter of patients in hospitals are at risk of under-nutrition or are already malnourished^(^ ^36^ ^)^. Under-nutrition has negative effects on treatment outcomes by increasing morbidity and mortality^(^ ^37^ ^)^. Nutritional care starts with systematic screening to identify ‘at risk’ patients and nutritional assessment and intervention planning to ensure that adequate nutritional support is provided at the right time. This approach is also reflected in a recently launched publication by the Action Group A3 of the European Partnership on Active and Healthy Aging of the European Commission^(^ ^38^ ^)^. The nutritional impairment identified by screening should therefore be relevant to objectives and outcomes individually determined, and interventions may vary according to individual assessment and care planning – for example, age or type of illness. In hospitals, special focus has to be given to acute and chronic illnesses, anthropometry, nutritional habits and history as well as individual therapeutic goals^(^ ^39^ ^)^.

The usefulness of malnutrition risk screening tools can be evaluated by a number of criteria. First, efficacy of a screening tool is of major importance. A hospital-based malnutrition risk screening tool should incorporate basic structural elements recommended by international scientific societies. ESPEN recommends four different variables to be included in a hospital nutrition screening tool^(^ ^40^ ^)^: anthropometric parameters, recent weight loss, individual food intake and its history and disease process and nutritional needs. The GMS tool addresses all the items recommended within the ESPEN Guideline for use in hospitals. Furthermore, the GMS was developed according to long-standing experience and local needs. Its novelty stems from the fact that it is very user-friendly and that it can be incorporated into any hospital IT platform, and therefore electronic patient record. It involves dietitians in an automated manner if malnutrition risk is detected in a patient screened by the staff on the wards. GMS therefore assures the multi-professional approach and quality control.

To address the screening tool’s statistical efficacy in terms of validity and sensitivity, 404 age-matched patients admitted to the internal medicine, orthopaedic or general surgery ward of the Graz University Hospital were screened in a blinded, randomised manner using the GMS, the NRS and the MNA-sf, respectively. Referring to the validation data of the screening tools used in terms of ‘gold standards’, one can state that the GMS tool is at least as sensitive in detecting an individual’s risk of malnutrition in patients admitted to an internal, surgical or orthopaedic ward. The results of this study indicate a high validity of GMS compared with malnutrition screening tools recommended for hospital use (Pearson’s *r* values of 0·78 according to the NRS and even higher, 0·84 compared with the MNA-sf).

In patients older than 70 years, the detection rate for risk of malnutrition using the GMS was quite comparable with results obtained using the NRS. This provides evidence for good criterion-related validity. Furthermore, accuracy of the GMS and NRS was 0·88, whereas accuracy value of the GMS and MNA-sf was 0·85. This indicates that there is a very high agreement between the GMS and these pre-existing validated nutritional screening tools. These findings are quite promising as several groups showed a rather fair agreement between MNA-sf and other screening tools such as the SGA in different cohorts^(^ ^40^ ^–^ ^42^ ^)^. Modification of SGA to distinct patient profiles in a large university hospital, specifically introducing ‘age’ as an independent risk factor, seems to raise the sensitivity of the GMS tool. Originally, the GMS had been designed for a general screening of all patients admitted to various hospital departments and wards. The sensitivity of the GMS tool seems to be comparable with the NRS, independent of patient age. According to data in our study, MNA-sf seemed to ‘overestimate’ risk of malnutrition when compared with NRS and GMS (independent of ward of admission and diseases). Young *et al*.^(^ ^43^ ^)^ argue in their publication that MNA-sf was designed to identify patients requiring further assessment with the MNA-long version, and highlighted the rather ‘poor performance’ of MNA-sf compared with SGA, pointing towards the different objectives of the MNA-sf and the SGA in that publication. This argument may also count for the comparison of the MNA-sf with the GMS in older patients in our study. However, differences in sensitivity between test systems did not reach the level of significance, at least in our study. Issues arising from this repetitive data from MNA-sf clearly include the question of feasibility of MNA-sf for the acute-care setting. Positive test scores for malnutrition risk screening highlight the need for further assessment of patients detected at risk. Although the MNA-sf was accurate, it identified a larger number of at-risk patients, as also reported by Raslan *et al.* ^(^ ^44^ ^)^, and therefore should be chosen only where healthcare services have sufficient resources to provide nutritional assessment and intervention programmes for all patients detected to be ‘at risk for malnutrition’. Similar arguments were also made by other authors^(^ ^42^ ^)^. Hospital management and clinicians should consider use of screening tools that are simple to implement and handle and tailor the screening and nutritional care process according to structures and resources available. Considered from these points of view, the GMS is a valid and easy-to-handle malnutrition risk screening tool, which may also be implemented in hospital IT systems.

Another factor influencing the efficacy of a screening test system is the reliability of the screening tool. In our study, we observed nearly ‘perfect’ results regarding equivalence and reliability of the GMS when compared with NRS and MNA-sf. Cohen’s *κ* values for the single items of the GMS ranged from 0·6 to 1·0, whereas the value of the total GMS scale was 0·82, which may be considered a highly sufficient reliability indicator for the test. Some items of the GMS scale showed higher inter-rater agreement values than others. Three items including BMI, severity of disease and age reached agreement of 1·0. These items are very ‘objective’ items, which vary only marginally or not at all as in the present sample. The measures of weight and height were performed in close time schedules between two raters. Data concerning changes in nutritional intake through loss of appetite, problems with chewing or swallowing or nausea/vomiting/diarrhoea were collected through interviewing patients. These subjective questions resulted in lower *κ* values from 0·60 to 0·63, which still reflects high agreement between the two raters. The blinded and randomised design of our study, including the activities of the various dietitians who carried out the interviews with the patients in the cohort of the GMS, underscores the effectiveness in terms of validity and reliability of the GMS as a malnutrition risk screening tool for daily practice in a large university hospital.

When introducing new tools into clinical practice, one also considers factors influencing effectiveness of test systems. It has been demonstrated long ago that awareness regarding malnutrition among hospital staff is rather low^(^ ^1^ ^,^ ^44^ ^)^. Up until now, these attitudes have not changed significantly. One reason for low acceptance of screening tools by professionals is lack of time. Nutritional screening is often seen as a time-consuming procedure with few consequences in a busy hospital. As effects of nutritional interventions are often not assessable during short periods of admission to acute care, NRS may be easily neglected. On the basis of the experience gained from several studies^(^ ^45^ ^,^ ^46^ ^)^, there seems to be a scarcity of nutritional knowledge among hospital staff and a lack of dietitians who are routinely present at the wards. It is therefore essential to develop a malnutrition risk screening tool with high accuracy in the prediction of individual malnutrition risk that is also easy to handle, and may therefore be accepted by most of the hospital staff. It must be easy to integrate into locally pre-defined work flows of the various hospital departments and must link dietitians into the patient-care process to optimise translation of information between different medical and healthcare professions working in the hospital setting. The GMS addresses all these requirements and is today well accepted by hospital staff as can be seen by the high professional fulfilment of the staff in routine day-to-day patient record keeping.

Using the current test setting, we could show prevalence for the risk of malnutrition in patients admitted to our university hospital, which is comparable with recent publications. An overall prevalence rate of about 30 % according to the GMS and 25 % according to the NRS goes along with previous clinical studies on prevalence of malnutrition in hospital inpatients. Higher prevalence rates in internal wards are also described in a range of publications as well as the fact that prevalence of malnutrition increases with age^(^ ^1^ ^–^ ^6^ ^)^. In our sample, malnutrition risk prevalence according to GMS was highest in age-group three (70+ years) with 41–45 % at risk compared with 26–29 % in age group two (45–70 years) and 19–22 % in age-group one (18–44 years). These data underline the importance of hospital risk screening tools for addressing malnutrition and the need to close inter-professional ‘gaps’ in the treatment plan and options in the acute-care setting. As a malnutrition screening tool refers only to the detection of ‘risk of malnutrition’ in patients admitted to the hospital, there is a great need for subsequent assessment and treatment. The use of risk screening alone does not necessarily result in improved outcomes unless there is a care pathway for malnourished patients or patients being at risk of malnutrition^(^ ^47^ ^)^. For this reason, the Nutrition Team of the University Hospital Graz developed an associated clinical pathway to standardise nutritional care in malnourished patients. In this concept, electronic data of patients screened positive for risk of malnutrition are transferred to a dietitian through the computer system. The set-up and implementation system chosen for risk screening strongly influence acceptance by healthcare professionals. Nowadays, nurses and doctors are accustomed to documenting their work electronically. A screening tool that is efficiently implemented into the hospital IT platform, and therefore individual care plan, will raise the acceptance of hospital staff as compared with the pencil and paper format^(^ ^17^ ^)^. It is therefore reasonable to expect that GMS will increase awareness of medical staff towards the challenge of malnutrition in hospitalised patients. Further studies will provide proof on that concept.

In conclusion, the GMS tool proves to be a valid and reliable instrument to detect risk of malnutrition in adult inpatients in acute-care hospitals. The GMS is easy to handle and provides reliable individual and overall results. GMS may be embedded in the hospital IT platform, and therefore provides short clinical pathways between different professions involved in the care process.
